# Nutcracker Syndrome Accompanying Pelvic Congestion Syndrome; Color Doppler Sonography and Multislice CT Findings: A Case Report

**DOI:** 10.5812/iranjradiol.11075

**Published:** 2014-05-15

**Authors:** Mikail Inal, Mihrace Yasemin Karadeniz Bilgili, Safa Sahin

**Affiliations:** 1 Department of Radiology, Kirikkale University, Yahsihan, Kirikkale, Turkey

**Keywords:** Hematuria, Renal Nutcracker Syndrome, Flank Pain

## Abstract

Nutcracker syndrome (NCS) is a rare pathology, caused by compression of the left renal vein (LRV) between the abdominal aorta (AA) and the superior mesenteric artery (SMA), due to reduction of the angle between AA and SMA. This leads to LRV varices, left gonadal vein varices and therefore, the pelvic congestion syndrome. For this reason, coexistence of NCS and pelvic congestion syndrome has been described. It manifests by hematuria, proteinuria, and nonspecific pelvic pain secondary to pelvic congestion, dyspareunia and persistent genital arousal. We report a 27-year-old woman who experienced hematuria and left flank pain. The diagnosis of NCS accompanied by pelvic congestion syndrome was missed initially, but later on the diagnosis was made by color Doppler ultrasound, abdominal computed tomography (CT) and CT angiography that were later performed. She refused interventional and surgical treatments, and was lost to follow up.

## 1. Introduction

The nutcracker syndrome (NCS) is compression of the left renal vein (LRV) by the abdominal aorta (AA) and the superior mesenteric artery (SMA), manifested by pain, hematuria, varicocele and lower urinary tract symptoms. NCS accompanying pelvic congestion syndrome is a rare pathology that can be easily missed by routine diagnostic methods.

## 2. Case Presentation

A 27-year-old woman was brought to the emergency department because of left flank pain, chronic pelvic pain and hematuria. She had genital complaints. She had no fever, vomiting, or diarrhea. She also denied any trauma to the abdomen or back. Her medical history was otherwise unremarkable. She had left flank tenderness. At that time, urinalysis showed numerous red blood cells. Hematuria persisted on multiple urinalyses.

**Figure 1.**
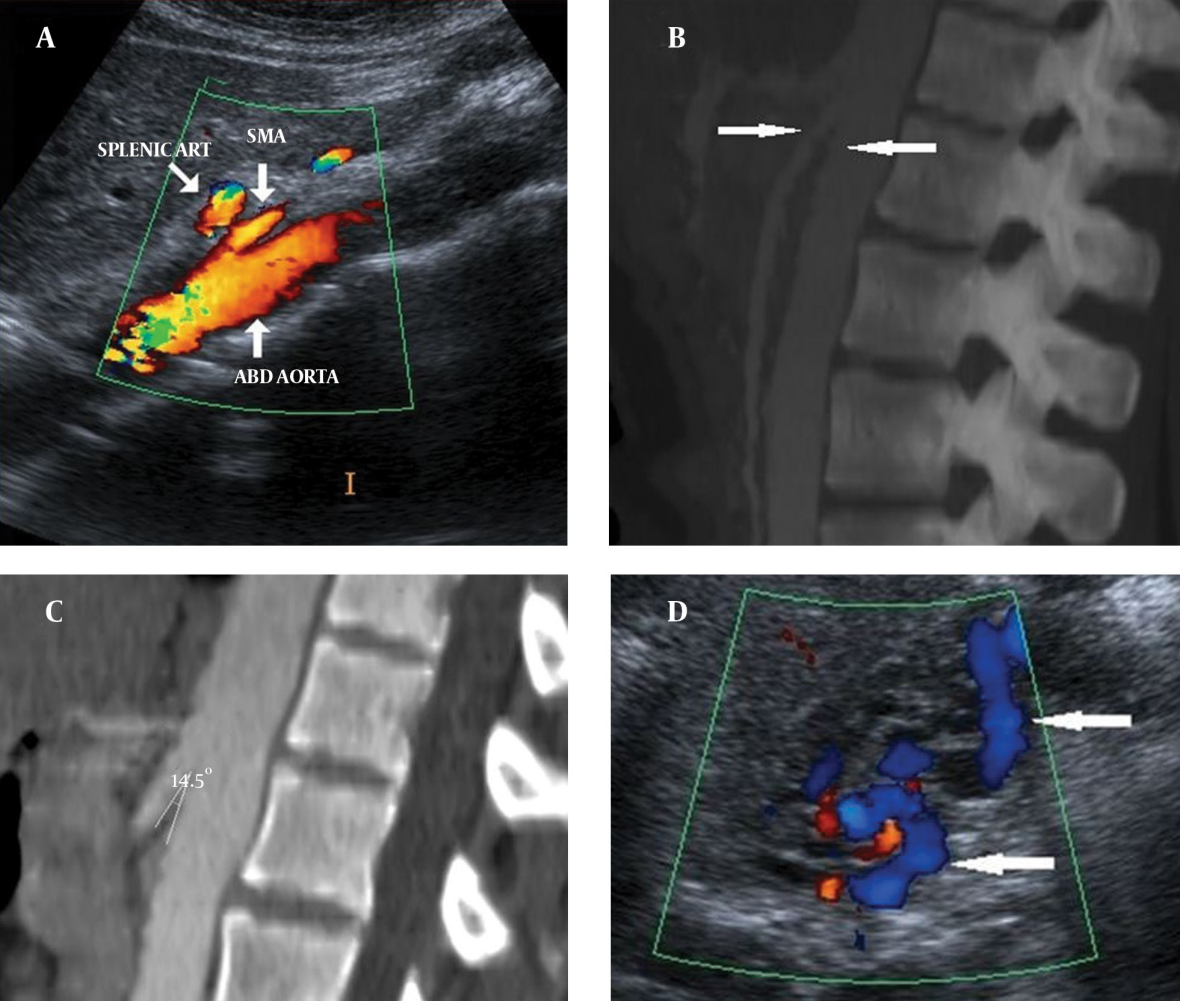
A 27-year-old woman with NCS and pelvic congestion syndrome A) CDUS and (B, C) sagittal CT images show reduction of the angle between AA and SMA (14.5°) (arrows). D) Pelvic CDUS shows pelvic varicose veins (arrows).

**Figure 2.**
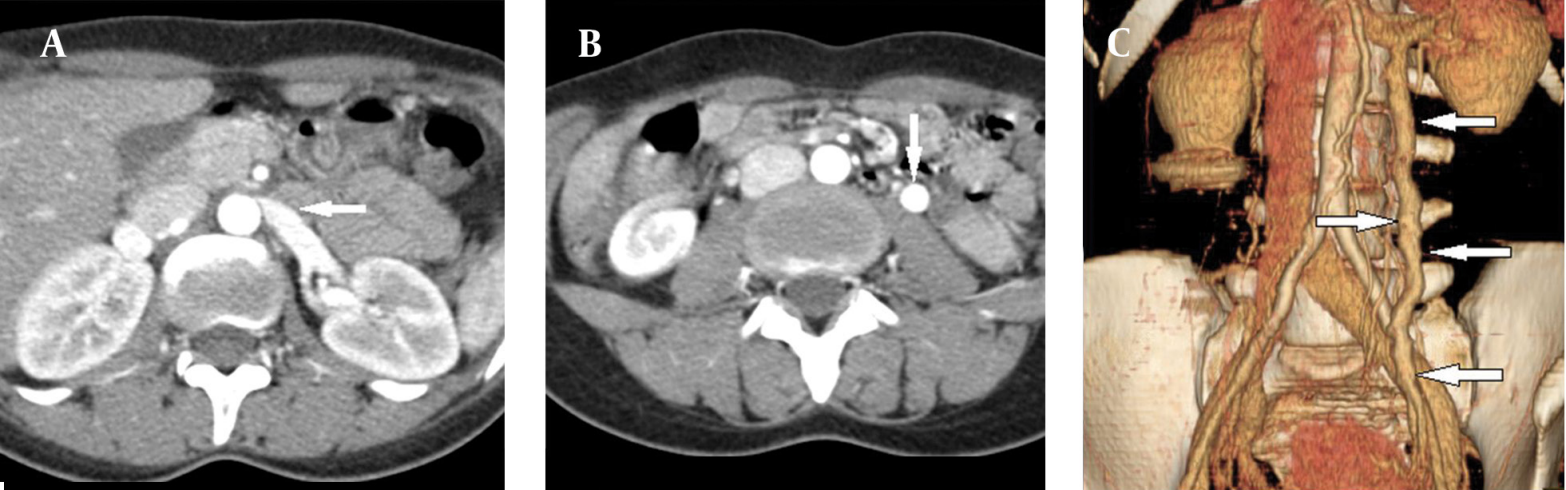
A) Axial CT image shows narrowing of the left renal vein between AA and SMA (beak sign) (arrow). B) Axial CT and C) Volume-rendered angiography images show retrograde filling of the left ovarian vein in the arterial phase consistent with reflux in pelvic congestion syndrome (arrows).

Color Doppler ultrasound (CDUS) and computed tomography (CT) images showed reduction of the angle between the AA and SMA. The angle between the AA and SMA was 14.5°. Pelvic ultrasonography (US), CDUS and CT images showed multiple pelvic varices including tortuous pelvic veins, dilated arcuate veins in the myometrium communicated with pelvic varicose veins (Figure 1). It was confirmed by CT that demonstrated compression of the left renal vein between the AA and SMA and also dilatation of the left ovarian vein attributable to NCS in axial and coronal oblique images. Axial CT reformatted oblique coronal images and volume-rendered multidedector CT (MDCT) angiography images showed retrograde filling of the left ovarian vein in the arterial phase consistent with reflux in pelvic congestion syndrome (Figure 2). The diagnosis of NCS accompanying pelvic congestion syndrome was established by these findings. With the consent of the patient and her parents, conservative treatment was decided upon.

## 3. Discussion

NCS is a rare, but treatable clinical condition (1). Because of the variability of symptoms and absence of consensus on diagnostic criteria, the exact prevalence of NCS is unknown, but it may be slightly higher in females (2). NCS is a well-known, but under-recognized cause of hematuria. The LRV normally crosses between the AA and SMA, where it may be compressed, resulting in a variable degree of LRV obstruction. This compression is called NCS (1). The normal angle between the SMA and the abdominal aorta is approximately 90° (3). The decreased angle between SMA and the aorta can cause NCS, this angle was reported as 39.3° ± 4.3° by Kim et al. and Fu et al. (4, 5). In our case, the angle between the aorta and the SMA was approximately 14.5°, which is nearly the lowest angle in comparison with previous studies and reported cases in the literature. On the other hand, NCS may also lead to LRV varices, left gonadal vein varices and therefore, the pelvic congestion syndrome (6). Pelvic congestion syndrome was first described by Richet in 1857 and Aran in 1858 (7). Pelvic varices can be seen in 10% of women in the general population and up to 60% of patients with pelvic varices may develop pelvic congestion syndrome. Pelvic congestion syndrome occurs mostly because of ovarian vein reflux, but it can also occur because of the obstruction of ovarian vein outflow resulting in reversed flow (8, 9).

Imaging, such as US, CT or magnetic resonance imaging (MRI), is required to diagnose NCS. However, the diagnostic procedure considered that the reference standard for establishing the diagnosis of NCS is invasive selective left renal phlebography with measurement of the pressure gradient between the LRV and inferior vena cava (1, 6, 10). Retrograde phlebography and cine video-angiography with reno-caval pressure gradient determination is accepted as the gold standard in establishing the final diagnosis of NCS. Cine video-angiography allows visualization of the point of compression of the left renal vein at the meso-aortic crossing and it also demonstrates peri-renal and peri-ureteral venous collaterals with reflux into the adrenal and gonadal veins and stagnation of the contrast in the renal vein. The normal reno-caval pullback mean gradient pressure ranges from 0 to 1 mmHg. The pressure gradient between the left renal vein and IVC should be higher than 3 mmHg to clinch the diagnosis of NCS (3). CDUS, CT, MRI and angiography all demonstrate LRV stenosis with proximal distention and the presence of collateral pathways in NCS (11). Narrowing of the LRV called the “beak sign” and CT findings can be useful for the diagnosis of NCS, and they reflect the pathophysiology of LRV compression between the SMA and the aorta (4). On the other hand, US is the initial diagnostic modality for the diagnosis of pelvic congestion syndrome. Combination of US and CDUS allow accurate diagnosis of pelvic varices. Several diagnostic criteria have been reported for pelvic congestion syndrome CDUS. These include tortuous pelvic veins with a diameter greater than 5-6 mm, slow (≈ 3 cm/s) or reversed flow, dilated arcuate veins in the myometrium communicating with pelvic varicose veins, and polycystic changes in the ovaries. Reversed flow in the ovarian vein can be suggested in the presence of ovarian vein filling on arterial phase CT images. Moreover, velocity encoded phase contrast MRI can be used to demonstrate ovarian vein velocity and flow direction. Selective ovarian venography has been accepted as the gold standard technique for the diagnosis of pelvic congestion syndrome (8, 12).

In the differential diagnosis of NCS and accompanying pelvic congestion syndrome, pathologies that cause pain and hematuria such as lithiasis, congenital vascular malformations, tumors, infections, parenchymal or urinary tract abnormalities, painful pelvic syndromes and organ or neighboring structure alterations must be excluded (6, 10). Supportive treatment is adequate if the symptoms are mild in NCS. Conservative treatment has been suggested for mild hematuria. Surgical or radiological interventions are indicated for severe pain, significant hematuria and renal functional impairment. Endovascular intervention is now considered the first line of therapy for management of this condition (1, 13, 14).

NCS accompanying pelvic congestion syndrome is a rare pathology that can be easily missed by routine diagnostic methods. It should be considered in the differential diagnosis of patients with hematuria, intermittent flank pain, nonspecific pelvic pain, dyspareunia, and persistent genital arousal without an apparent cause. An inclusion of this entity in the differential diagnosis of patients with an unknown cause of hematuria and flank pain can achieve efficient management for this condition.
